# Improving the Quality of Transurethral Resection of Bladder Tumour (TURBT) Operative Notes Following the European Association of Urology Guidelines: A Completed Audit Loop Study

**DOI:** 10.7759/cureus.30131

**Published:** 2022-10-10

**Authors:** Daniel N Guerero, Angus Bruce, Sushanth Vayalapra, Vishnu Menon, Mohammed El Hadi, Shehab Khashaba

**Affiliations:** 1 Trauma and Orthopaedics, Russells Hall Hospital, Dudley, GBR; 2 Urology, Sandwell and West Birmingham NHS Trust, Birmingham, GBR; 3 Urology, King Hamad University Hospital, Muharraq, BHR

**Keywords:** guideline, clinical documentation improvement, non-muscle invasive bladder cancer, operation note, turbt

## Abstract

Background

The European Association of Urology (EAU) recommends that the operative steps and documentation necessary for successful and appropriate management of bladder cancer include identifying factors necessary to assign disease risk stratification, clinical stage, adequacy of resection and the presence of complications and immediate intravesical chemotherapy administration.

Aim

To assess and improve the adequacy of current transurethral resection of bladder tumour (TURBT) documentation at a district general hospital in the UK against the EAU 2022 guidelines.

Methods

Operative notes over a one-year period were assessed for the inclusion of key steps to achieve a comprehensive TURBT as outlined by EAU guidelines. Outcomes included documentation on the details of the operative findings and intervention as well as the perioperative assessment. A standardised template for TURBT procedures was created and surgical staff received training on its usage. The audit was subsequently repeated after six months to assess for improvements.

Results

TURBT documentation of 78 cases in the first cycle was compared to 37 cases from the second cycle. Significant improvements in the documentation of tumour size (46% to 89%; p<0.05), tumour description (59% to 89%; p <0.05), depth of resection (36% to 89%; p<0.05), administration of chemotherapy (21% to 46%; p<0.05) and assessment for perforation (22% to 68%; p=0.001) were demonstrated. Improvements in pre-operative and post-operative examination rates under anaesthesia also achieved statistical significance (47% & 14% respectively to 89%; p<0.05). There was an increase in the documentation of completeness of resection but this did not achieve statistical significance (59% to 68%; p=0.42).

Conclusion

The operative note template led to the improvement in the documentation, improving the risk stratification of bladder cancer in patients undergoing TURBT. The use of procedure-specific operative note templates should be adopted for all commonly performed procedures to improve the completeness of documentation.

## Introduction

The purpose of an operation note is to facilitate the continuity of care as well as provide a medico-legal record of a patient’s care [1]. The Royal College of Surgeons (RCS) has produced guidance entitled “Good Surgical Practice” which indicates that there should be a clear, preferably typed, operative note for every procedure [2]. The accuracy and completeness of operative documentation have significant implications on patient safety, research, education and medico-legal proceedings. Patient safety can be affected by the quality of documentation when there is a transfer of patient care, either immediately after the procedure during the uro-oncology multidisciplinary team discussion or much further in the future in the context of disease recurrence. Operative documentation has been reported to be inadequate across surgical specialties with previous reports indicating that up to 45% of operative notes are indefensible from a medico-legal point of view [3].

Bladder tumours are typically managed using a multimodal approach to intervention [4]. Transurethral resection of bladder tumour (TURBT) is a key aspect in the initial diagnosis and further management of these tumours as it facilitates assessment for muscle invasion, degree of differentiation, histological type and the number of lesions. The aim is to remove all visible tumours, down to the muscle and make an accurate diagnosis via pathological analysis. Bladder cancer recurs frequently and is ranked as one of the most costly malignancies to treat. The risk of recurrence is based on patients’ age, the operative note documentation, the histological assessment and diagnosis of the depth of muscle penetration on the pathological specimen. All the information is discussed in an uro-oncology multi-disciplinary team (MDT) meeting and subsequent interventions are based on the information collected from the operative notes and the histological assessment. These follow-up discussions and their resultant decisions can be significantly influenced by inaccurate or incomplete documentation as they rely heavily on the details of the TURBT operative note.

Thomas et al. [5] first reported the notion of having an agreed minimal dataset for documentation in urological practice and demonstrated that it was possible via the development of several data capture forms through an iterative process with urology consultants. However, this study primarily focused on the standardisation of documentation related to the initial and continuous clinical assessments. Dukic et al. [6] reported improvements in urology operative notes through the use of software for electronic documentation but focused on meeting the basic non-specific criteria as set out by RCS England for operative notes of all surgical specialties.

This quality improvement project was designed to assess procedure-specific operative note details after recognition of deficiencies at two local uro-oncology MDT meetings. The study aimed to assess how the current practice of operative note documentation measured against the recommendations from the European Association of Urology (EAU) Guidelines.

## Materials and methods

This study was based on the practice of one Urology department operating across two District General Hospitals, serving a population of 530,000 [7].

A list of patients who potentially underwent TURBT was constructed through collaboration with the local bladder cancer patient tracker, uro-oncology specialty nurses and hospital coders for CPT code (51520, 51525, 51530) and bladder cancer ICD-10 code (C67). The notes of all patients were reviewed by two independent reviewers and patients were included in the study if they had a TURBT procedure between January 2019 and January 2020. This included patients who were having their first TURBT as well as any subsequent redo TURBT procedures.

The operation note of each included patient was assessed for the inclusion of the recommended necessary operative steps to achieve a comprehensive TURBT. These steps were based on the EAU guidelines and included documentation on the 1) Details of the operative findings (number of tumours, tumour size and tumour characteristics), 2) Details of the operative intervention (completeness of resection, depth of resection, administration of immediate dose of chemotherapy) and 3) Details of the perioperative assessment (assessment for perforation, pre-operative and post-operative examination under anaesthesia). The date of the procedure and name as well as the career grade of the surgeon writing each operative note were also collected.

**Figure 1:**
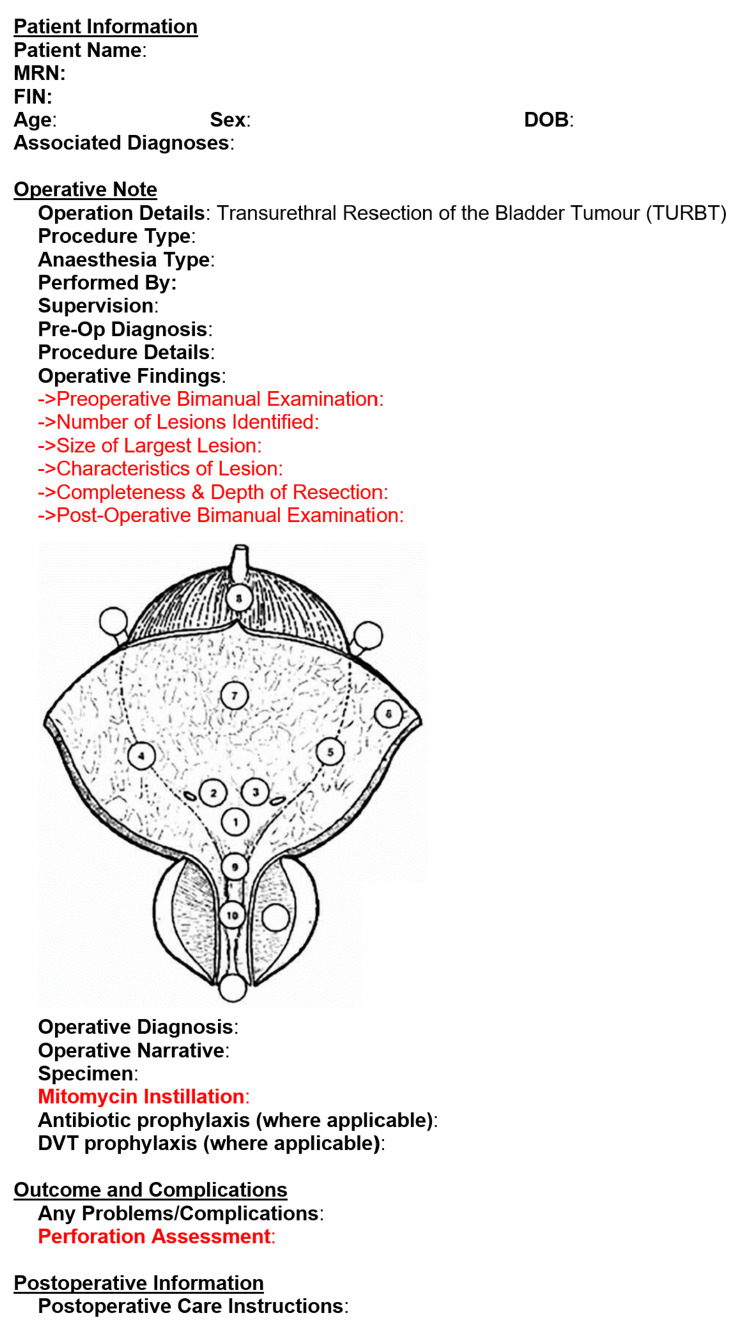
The TURBT Operative Note Template

The data from the first cycle was presented to the department and senior Urology staff (registrar and consultants) noting the deficiencies. A standardised TURBT Operative Note Documentation Template was created to include all the fields necessary for a standard TURBT documentation note, as well as the standard required fields as directed by the RCS (see Figure 1). Operating surgeons received one-to-one training on the utilization of the template.

The audit was subsequently repeated with the inclusion of further patients who underwent TURBT between May and October 2020.

The software SPSS v25.0 (IBM Corp., Armonk, NY) was used for data analysis. Descriptive statistics were used to compute the frequencies and percentages. The Fischer’s exact test was used to compute the significant changes in the frequencies. All the tests were two-sided and a p-value < 0.05 was considered statistically significant.

## Results

A total of 103 patients were included in the original compiled list of patients who potentially had a TURBT procedure. Twenty-five patients were excluded due to one or more of the following reasons: refusal of operative management, operative management deemed inappropriate, inaccessible documentation or procedure cancellations. Seventy-eight patients were found to have had TURBT between January 2019 and January 2020 and were included in the first cycle of the audit. The second cycle included a further 37 patients who underwent TURBT between May and October 2020.

The average age of patients included in the first cycle was 72 (range 41-90) whereas the cohort included in the second cycle was marginally younger with an average age of 67 (range 31-89). Overall, both cycles had a similar sex distribution with the first cycle being 76% male vs 78% in the second cycle. A greater proportion of the notes in the second cycle were written by Consultants as opposed to Registrars (see Table 1).

**Table 1:** Demographic details of the patients included in the study.

Case Details	Cycle 1 Documented n (%)	Cycle 2 Documented n (%)
Patient Age	72 (41-90)	67 (31-89)
Male	59 (76%)	29 (78%)
Female	19 (24%)	8 (22%)
Consultant Notes	19 (24%)	23 (62%)
Registrar Notes	59 (76%)	14 (38%)

There were significant improvements in the rate of documentation of key operative steps in the second cycle when compared to the first. Documentation of the number of tumours remained perfect (100%) across both cycles with the majority of cases reported to only have one tumour in both cycles (Cycle 1: 67% vs Cycle 2: 70%) (see Table 2).

**Table 2:** Number of tumours reported in each cycle.

Number of Tumours	Cycle 1 n (%)	Cycle 2 n (%)
1	52 (67%)	26 (70%)
2	11 (14%)	2 (5%)
3	2 (3%)	1 (3%)
4	2 (3%)	1 (3%)
5-10	3 (4%)	0 (0%)
Multiple (10+)	8 (10%)	7 (19%)

Statistically significant improvements in the documentation of tumour size (46% to 89%; p<0.05) and tumour description (59% to 89%; p<0.05) were observed. The most commonly reported tumour size across both cycles was 1-2 cm (see Table 3) with papillary being the most commonly reported tumour descriptor or characteristic (see Table 4). Improvements in documentation of depth of resection (36% to 89%; p<0.05), administration of chemotherapy (21% to 46%; p<0.05), assessment for perforation (22% to 68%; p=0.001) and pre/post-operative examination under anaesthesia (47% & 14% respectively to 89%; p<0.05) were also observed (see Table 5).

**Table 3:** Tumour sizes reported in each cycle.

Tumour Size/cm	Cycle 1 n (%)	Cycle 2 n (%)
<1cm	10 (13%)	8 (22%)
1-2cm	13 (17%)	12 (32%)
2-3cm	10 (13%)	7 (19%)
3-4cm	1 (1%)	3 (8%)
5cm	2 (3%)	3 (8%)
Undocumented	42 (54%)	4 (11%)

**Table 4:** Tumour characteristics reported in each cycle.

Tumour Descriptor	Cycle 1 n=78 (%)	Cycle 2 n=37 (%)
Solid	10 (13%)	10 (27%)
Papillary	23 (30%)	18 (49%)
Superficial	8 (10%)	2 (5%)
Necrotic	4 (5%)	3 (8%)
Muscle Invasive	1 (1%)	0 (0%)
No Descriptor	32 (41%)	4 (11%)

**Table 5:** Rates of documentation of key TURBT operative steps across both cycles. TURBT: Transurethral Resection of Bladder Tumour

Measured Parameter	Cycle 1 Documented n (%)	Cycle 2 Documented n (%)	P-values
Number of Tumours	78 (100%)	37 (100%)	1.00
Tumour size	36 (46%)	33 (89%)	<0.05
Tumour description/characteristics	46 (59%)	33 (89%)	0.001
Completeness of resection	46 (59%)	25 (68%)	0.42
Depth of resection	28 (36%)	33 (89%)	<0.05
Administration of Chemotherapy	16 (21%)	20 (46%)	<0.05
Perforation assessment	17 (22%)	25 (68%)	0.001
Pre-operative Examination Under Anaesthesia	37 (47%)	33 (89%)	<0.05
Post-operative Examination Under Anaesthesia	11 (14%)	33 (89%)	<0.05

There was an improvement in the documentation of completeness of resection but this did not achieve statistical significance (59% to 68%; p=0.42) (see Table 5). Eight of the patients (22%) in the second cycle had a comprehensive operative note by EAU standards while only one (1%) had the same in the first cycle.

## Discussion

This study assessed the completeness of operative notes for an important, common procedure in general urological practice. The contents of operative notes are of significance due to their implications for post-operative care and potential medico-legal proceedings. There is an added layer of importance for cancer procedures as these cases typically require an extensive multidisciplinary approach in the perioperative phase. There is also a risk of disease recurrence at a point far removed in time from the index operation, in which case accurate and complete historical documentation may prove to be invaluable through altering future management.

The RCS stipulates that surgeons must keep “accurate, comprehensive, legible and contemporaneous records” [2]. Their ‘Good Surgical Practice’ guidance indicates that operative notes should be clear and preferably typed. They should include the following fields: date, time, the urgency of the procedure, name of operating surgeon and anaesthetist, the operative procedure carried out, incision, operative diagnosis and findings, issues/complications, any extra procedures performed, and tissue samples taken or altered, identification of any prostheses, details of closure technique, anticipated blood loss, antibiotic prophylaxis decision, deep venous thrombosis (DVT) prophylaxis decisions, post-operative care instructions and signature [2]. The EAU guidelines for the management of non-muscle invasive bladder cancer detail the necessary operative steps for a successful TURBT. The template created and used in this study was designed to fulfill the guidance outlined in both key documents and was integrated into the hospital’s electronic medical records (EMR).

The use of EMRs provides prompts for clinical actions and automated measures of quality outcomes [8]. EMR facilitates the collection of data in normal clinical practice in a way that can be easily represented graphically for presentation and easily accessed for research purposes. A 2014 poll of physicians using EMRs found that 65% indicated that patient care had improved while only 5% indicated a negative effect on the quality of care provided [9]. There is an added benefit of improved relationships between teams within an organisation and externally as communication between teams is significantly improved with the improved legibility and access that digital records provide [10].

EMRs have an operation note template that meets the criteria defined by the RCS for an operative note and can be customized. The adapted template remained familiar to the users and provided prompts for procedure-specific data points as outlined by the EAU guidelines. The study template was readily accessible to surgeons when writing their operative notes only requiring one extra click from their normal practice. This ease of access and familiarity made this method of intervention more suitable than alternatives such as the use of memory aids in orthopaedic and ENT theatres as reported by Mustafa et al. [11] and Shayah et al. [12].

Coughlan et al. [13] reported a study with a similar concept where a typed proforma was used for orthopaedic operations and included speciality-specific headings such as tourniquet time. However, a study by Shah et al. [14] most closely resembled our study as they created procedure-specific electronic templates and circulated these to the department. However, both studies required surgeons to access a shared drive to look at the template for editing with subsequent printing of the templates. These studies were limited as they could not integrate the template within an EMR system. Abbas et al. [15] also performed a similar study in general surgery with a customised template for laparoscopic appendicectomies. A procedure-specific proforma was created with fields for entry of data on port usage (number, location, type) and the characteristics of the appendix. However, the focus of data collection was on compliance with standard RCS operation note requirements and no data were presented on the effect on documentation of key procedure-specific details.

Senior surgical staff in this study also moved on to create templates for other commonly performed procedures such as Flexible Cystoscopy, Transurethral Resection of the Prostate, Ureteroscopy and Laser Lithotripsy/Ureteric Stent insertion after receiving training on the usage and integration of the study template. Most EMRs have templating features and as such the effect demonstrated in this study should be easily transferable across hospitals with EMRs. The discussion on the contents of a comprehensive operative note prompted by this study resulted in improved compliance in other operations. Singh et al. [16] demonstrated this effect when it was reported that documentation can be improved by increasing awareness of current deficiencies in documentation.

Two studies have previously reported methods of improving the completeness of TURBT documentation. Anderson et al. [17] designed and implemented an intra-operative checklist of key steps to achieve a comprehensive TURBT whereas Haddad et al. [18] performed a training session for residents on comprehensive TURBT operative reporting and provided a checklist for dictating. Both studies noted significant improvement in the reporting of critical elements in operative note documentation. The key similarity between these studies and this current study is that key elements for a comprehensive TURBT procedure were identified and highlighted to the surgical staff. However, the approach taken to achieve this was different in each study. A further study implementing all of these methods should be conducted to assess for a potential cumulative benefit when utilised in one department.

Limitations of this study included the small sample size in the re-audit period. This reduction in completed procedures was largely due to the disruption of services during the coronavirus pandemic. Another potential for bias in this study is the significant increase in the proportion of operative notes being completed by consultants in the second cycle. This may have contributed to the improvement in operative note documentation reported across cycles.

## Conclusions

The results of this study showed improvement in the documentation of most of the key steps of the TURBT procedure. Even though electronic record-keeping is not available to all hospitals, some of the beneficial effects on completeness of documentation should be translatable to any practice by simply making printed custom operative note templates available for common core procedures.

In recent years there has been a move towards the use of electronic templates for operative documentation which has shown great improvements in documentation, legibility and completeness as defined by RCS standards. Future development should focus on a move towards speciality and procedure-specific documentation to take into account the importance and variability of speciality and procedure-specific details. This seems feasible in the future given the National Health Service's goal to become completely digital over the coming few years.
